# Photoperiodic and circadian bifurcation theories of depression and mania

**DOI:** 10.12688/f1000research.6444.1

**Published:** 2015-05-06

**Authors:** Daniel F. Kripke, Jeffrey A. Elliott, David K. Welsh, Shawn D. Youngstedt

**Affiliations:** 1 Department of Psychiatry and Center for Circadian Biology, University of California, San Diego, CA, 92093-0603, USA; 2 College of Nursing and Health Innovation, Arizona State University, Phoenix, AZ, 85004-4431, USA

**Keywords:** depression, mania, bipolar disorder, circadian rhythm, suprachiasmatic nucleus (SCN), photoperiod, triiodothyronine (T3), thyrotropin (TSH)

## Abstract

Seasonal effects on mood have been observed throughout much of human history.  Seasonal changes in animals and plants are largely mediated through the changing photoperiod (i.e., the photophase or duration of daylight).  We review that in mammals, daylight specifically regulates SCN (suprachiasmatic nucleus) circadian organization and its control of melatonin secretion.  The timing of melatonin secretion interacts with gene transcription in the pituitary pars tuberalis to modulate production of TSH (thyrotropin), hypothalamic T3 (triiodothyronine), and tuberalin peptides which modulate pituitary production of regulatory gonadotropins and other hormones.  Pituitary hormones largely mediate seasonal physiologic and behavioral variations.  As a result of long winter nights or inadequate illumination, we propose that delayed morning offset of nocturnal melatonin secretion, suppressing pars tuberalis function, could be the main cause for winter depression and even cause depressions at other times of year.  Irregularities of circadian sleep timing and thyroid homeostasis contribute to depression.  Bright light and sleep restriction are antidepressant and conversely, sometimes trigger mania.  We propose that internal desynchronization or bifurcation of SCN circadian rhythms may underlie rapid-cycling manic-depressive disorders and perhaps most mania.  Much further research will be needed to add substance to these theories.

## Review and theoretical interpretation

In this presentation, we review the seasonality of mood disorders and the photoperiodic control of seasonality among mammals, in order to present new theories of the causes of depression and mania. The seasonal timing of daily light exposures reorganizes cellular circadian clocks in the bilateral suprachiasmatic nuclei (SCN) of the hypothalamus above the optic chiasm to regulate the evening rise and duration of melatonin secretion ^1– 4^. In mammals, an exquisite mechanism in the pars tuberalis (PT) interprets daylength (photoperiod) from the duration of nocturnal melatonin secretion and particularly from the early morning termination time of secretion. Melatonin offset influences thyrotropin (TSH) and tuberalin hormone synthesis by the PT ^5, 6^. These PT products then influence pituitary hormones and induce deiodinase 2 (DIO2), increasing hypothalamic triiodothyronine (T3), which maintains seasonal gonadal fertility. We propose that this same mechanism may be a key controller of mood. Inadequate hypothalamic T3 may cause depression, whereas excessive hypothalamic T3 may mediate mania. Peculiar aspects of the induction of mania by bright light or sleep restriction suggest a theory that mania may be promoted by bifurcation of the circadian phasing of neuronal firing in two distinct populations of SCN neurons. Finally, we will propose particular inquiries where more research is needed to explore and validate these theories.

## Mood and seasonality

To understand mood disorders, we must hope to understand seasonality. Associations of mood changes with the seasons have been observed since antiquity ^7^. Likewise, seasonality in suicide was recognized by the ancients and has been studied with modern scientific methods for over a century ^8– 10^. In the short dark days of winter, many people, especially bipolars (people who have experienced mania or hypomania) ^11^, experience a tendency towards low mood, usually mild. Winter, however, may not be the season for the most serious manifestations of depression ^9^. The seasonal peaks for suicide and for hospital admissions for depression are in April or May in many Northern Hemisphere data sets. Additionally, peaks in mania are often observed in May or June, and both depression and mania sometimes express secondary peaks in the fall ^7, 11– 13^.

In small mammals in temperate climates, a quiescent interval or even hibernation in winter may be followed by a spring mating season sometimes highlighted by increased venturesome wandering or migrations, increased aggressiveness, rutting behaviors among males, and ovulation and mating receptivity among females. Perhaps these winter and spring behaviors resemble some aspects of depression and mania, respectively. According to Wehr *et al.*, seasonality in primates is quite variable and seems to be influenced by complexities of food availability, latitude, and body size: since in tropical and equatorial environments, the rainy season may be more influential than temperature or day length ^14^. Many human groups have a peak in conception close to the spring equinox, sometimes with a secondary peak in the fall or at Christmas ^15, 16^. Perhaps human populations have become variable partly because human groups have moved to new latitudes and climates without much time for evolutionary adaptation ^17^. Moreover, seasonal reproductive trends have tended to flatten as modern lighting and heating became available ^15^. Because of the wide range of environmental adaptations and recent migrations, human populations may have diversities in seasonal behaviors and mood more complex than common mammalian models.

## Photoperiodic and molecular control of seasonal responses through melatonin

In mammalian species as diverse as hamsters and sheep, seasonal behaviors are largely regulated by the photoperiod: the interval of daylight within each 24 hours, also called the photophase ^2, 18^. Gross locomotor activity varies with photoperiod: for example, among nocturnal rodents, activity is compressed into the short nights of summer, but expands in duration as nights grow longer in winter. The photoperiod regulates seasonal responses specifically through SCN control of nocturnal pineal melatonin secretion. Melatonin generally increases after dusk and terminates by dawn, under control of the SCN circadian timing system as regulated by day length ^4^. It has been shown that in mammals, pineal melatonin secretion is under control of a multisynaptic pathway arising primarily from the dorsomedial SCN AVP cells. The interval of melatonin secretion is short during the short nights of summer and longer during the long nights of winter in most animals, whether nocturnal or diurnal. Melatonin has been considered a neuroendocrine signal of the night or scotophase (the dark inverse of the photophase). There is evidence that nocturnal melatonin secretion feeds back on SCN neurons to modulate certain components of the circadian molecular clockwork ^19^. The interval of nightly locomotor activity among nocturnal rodents and the interval of sleep propensity among diurnal adult humans both correspond roughly, but not exactly, to the interval of melatonin secretion by the pineal. A wealth of studies suggest that it is the duration of the photoperiod-regulated nocturnal melatonin secretion that controls seasonal increases in gonadal size and the adaptive timing of mammalian breeding activities ^20^.

Whether mammals are nocturnal or diurnal in activity, most SCN neuronal firing occurs during the day. High-level multiunit firing expands in duration in long days. Thus, in diurnal animals, multiunit activity and waking physical activity tend to occur together, whereas among nocturnal animals they are inverse.

A useful hypothesis has been that two coupled circadian oscillators interact to regulate nocturnal activity, e.g., in rodents: an evening oscillator (E) has been linked to the burst of locomotor activity beginning about dusk and a morning oscillator (M) may be primarily responsible for timing the cessation of locomotor activity before dawn ^20^. In theory, the E and M oscillators spread apart during long nights, allowing an increased span of nocturnal locomotor activity in winter, whereas in summer, the long hours of daylight and short nights squeeze the interval between E and M, resulting in a shorter duration of nocturnal locomotor activity. Likewise, the evening increase and morning decline of melatonin secretion seem to be influenced by the separate timing of evening and morning oscillators, i.e. their phase-timing relationships ^20^. The expansion of the nocturnal interval between E and M varies inversely with the compression of the diurnal interval of rapid SCN neuronal firing, and vice versa. Perhaps nocturnal locomotor activity and melatonin secretion are inhibited by daytime SCN neuronal firing.

Recent work in nocturnal rodents has revealed that these E and M oscillators are embodied in groups of coupled neurons located in the SCN ^3, 21, 22^. The daylight photoperiodic input, sensed mainly by intrinsically blue-light-sensitive retinal ganglion cells ^23^, is transmitted by their axons to a ventrolateral and largely rostral “core” region of each SCN, where a key neurotransmitter is vasoactive intestinal polypeptide (VIP) ^24^. The core neurons send VIP axons to surrounding dorsomedial “shell” regions (mainly caudal), entraining the shell neurons, which then transmit arginine vasopressin (AVP) signals to other regions such as the hypothalamic paraventricular nucleus, as well as feeding back on the core ^25^. It might seem plausible that the core would encompass the evening oscillator and the shell the morning oscillator ^26^, yet recent studies suggest a more complex tri-dimensional distribution of cell groups ^27^. From another perspective, the most caudal SCN cells seemingly correspond to the morning oscillator ^28^, and some of the rostral cells correspond to the evening oscillator ^21, 29^, but there is at least one additional cell group in the rostral SCN region which may be more closely linked to shell than core ^21, 30, 31^. Under the influence of long photoperiods (short nights), the caudal morning oscillator tends to phase advance several hours, drawing closer to the rostral evening oscillator ^32^, as the interval of behavioral activity of nocturnal rodents is compressed, but inversely, daytime intense SCN neuronal firing expands in duration. There seems to be greater spatial and neuropharmacologic complexity than the rostral-caudal or core-shell dichotomies suggest, and there is insufficient evidence to firmly link particular SCN neuronal populations to E and M or particular features of motor activity and melatonin secretion. Also, there may be differences among species. Unfortunately, many of the studies of SCN responses to photoperiod have been conducted in laboratory-bred mice that do not synthesize melatonin (and therefore, lack melatonin feedback upon SCN neuronal phases).

During the night, melatonin can be suppressed acutely by light. Rather dim light will suppress melatonin among nocturnal rodents, and brighter (but still dim) light will likewise shift circadian phases in nocturnal rodents ^33, 34^. Much brighter light, brighter than most contemporary indoor illumination, is usually required to suppress melatonin in humans ^35^, and even brighter light resembling sunlight or bright cloud cover is required for maximally strong resetting of the human clock through circadian phase shifting ^36^. However, there are exceptions to the rule that bright light is required for melatonin suppression and phase shifting in humans, perhaps related to nocturnal dark adaptation of the eyes ^37– 39^. Effects of dawn simulation during sleep (with closed eyes) may imply that the circadian system is especially sensitive to light towards the latter half of nocturnal sleep when the greatest retinal dark adaptation might have occured ^37^. Moreover, the phase-shifting sensitivity of the hamster phase-response curve is modified by long and short photoperiods ^40^, and the same might be true in humans.

## Melatonin regulation of molecular biology in the pars tuberalis region

**Figure 1.**
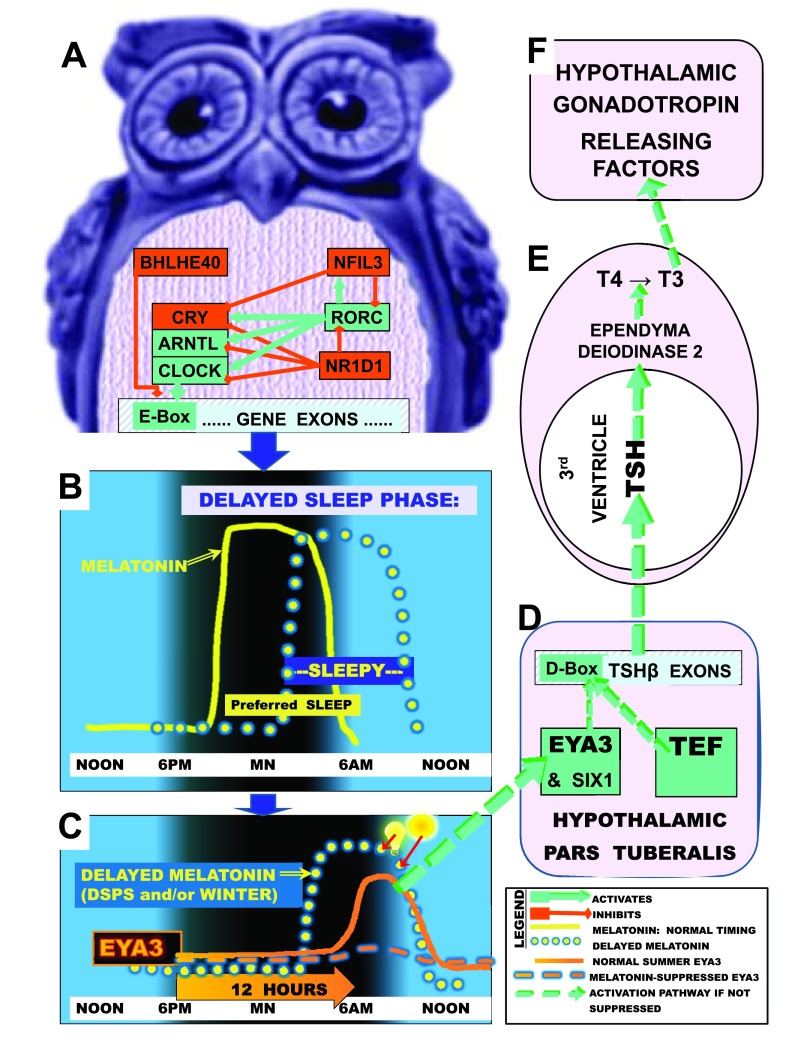
Delayed sleep phase (DSP) and photoperiodic disturbances. **A**, Depicted is some of the circadian gene network that times transcription through pathways leading to E-box activation (green) or which deactivates transcription and E-box promoter action (red) in a night owl or depressed person. **B**, The yellow line illustrates normal melatonin secretion commencing shortly before the preferred nocturnal sleep time and terminating about the time of awakening near dawn, so that preferred sleep times and sleepiness normally correspond. The yellow dotted line illustrates how in DSP, melatonin secretion offset may become delayed, with correspondingly delayed sleep propensity. **C**, The gene *EYA3* reaches a sharp peak in pars tuberalis transcription about 12 hours after darkness onset (solid orange line), but if melatonin is still elevated (due to long nights of winter, long time in bed, or DSP), the EYA3 peak is largely suppressed (dashed orange line). Bright lights (light bulb and sun symbols) conversely suppress and advance melatonin offset (red arrows), disinhibiting EYA3. **D**, After short nights in summer, EYA3, SIX1 and TEF coactivate near a D-box on the *TSHB* promoter. TSHB hybridizes with TSHA, releasing active TSH into 3 ^rd^ ventricle CSF ^6, 44^. **E**, TSH circulates retrograde to promote DIO2 which converts T4 to T3. **F**, T3 promotes synthesis and release of gonadotropin hormones, implementing summer reproduction and good mood. Revised with permission from Kripke *et al.*, *Psychiat. Invest.*, 2014 ^99^.

The duration of nocturnal melatonin secretion regulates seasonal gonadal growth and breeding through hypothalamic regulation of the most active thyroid hormone, T3 (triiodothyronine); as will be discussed, T3 is likewise crucial to mood. The importance of T3 in photoperiodic control was recognized in Japanese quail ^41^ and then confirmed in mammalian species. In mammals, melatonin binds to a dense supply of melatonin receptors in the pars tuberalis (PT) in the rostral anterior pituitary just below the hypothalamic median eminence ^42^. A primary effect of melatonin in PT is control of the transcription factor *EYA3* ( Figure 1). In the summer when the interval of melatonin secretion ends early, *EYA3* is strongly transcribed in PT in the early morning about 12 hours after dark, a time when circulating melatonin is low ^43^. While TEF binds to a D-Box motif on the *TSHB* promoter in PT, SIX1 binds to an adjacent So1 site on the promoter, and EYA3 binds either to SIX1 or to a nearby site on the *TSHB* promoter ^5, 6^. Together, EYA3, SIX1, and TEF combine to promote pars tuberalis transcription of the *TSHB* gene. *TSHB* transcription leads to translation of the thyroid stimulating hormone beta chain, which hybridizes with the TSHA polypeptide to form the active dimer, thyroid stimulating hormone (TSH). PT TSH then passes retrograde into the 3 ^rd^ cerebral ventricle CSF ^5, 6, 18, 44^. Very high local concentrations of TSH in the 3 ^rd^ ventricle bind to TSH receptors on ependymal tanycytes lining the ventricular surface, which in turn promotes transcription of a deiodinase (DIO2) that converts T4 to T3, especially in the tanycytes. This produces high concentrations of T3 in the third ventricle and adjacent hypothalamic region, close to TRH (thyrotropin releasing hormone) cells which homeostatically respond to T3 feedback ^41, 43, 45^. Since T3 passes into the brain poorly, most brain T3 is produced within the brain and substantial portions by these 3 ^rd^ ventricle tanycytes ^46^. PT production of TSH is not influenced by homeostatic feedback from TRH and T3, and unique PT glycosylation of TSH prevents the small amounts of TSH produced by PT from directly influencing the thyroid ^47^.

T3 promotes the secretion of GnRH into portal blood, leading to increased pituitary release of LH and FSH, which augment testosterone, estrogens, and progesterone, thus promoting seasonal reproduction ^43, 48^. Melatonin is thought to suppress prolactin secretion, either through direct effects on the PT transmitted to the anterior pituitary through PT tuberalin peptides or through actions of CSF T3 upon hypothalamic TRH and dopamine secretion ^49^, which then influence pituitary prolactin secretion ^42, 50– 52^. Interestingly, the PT tuberalins derived from the gene *TAC1* may promote pituitary ACTH, GH, TSH, LH and FSH secretion, as well as influencing prolactin ^52^.

During the long nights of winter, melatonin may remain elevated during those early morning hours when maximal *EYA3* transcription is scheduled. Because in winter, elevated morning melatonin inhibits PT *EYA3* and *TAC1* transcription, PT TSH production is inhibited, thus reducing expression of DIO2, production of T3 by tanycytes, and ultimately inhibiting gonadal maintenance in winter. Note that in humans, reduced hypothalamic T3 would lead to loss of libido, a major component of depression. Conversely, increased libido is a typical component of mania.

Although sheep are autumn short-day breeders, much of the melatonin control of T3 among rodents and sheep is similar ^5, 6, 18, 44, 53^. Almost all mammals, both long-day and short-day breeders, produce more prolactin in the summer. Humans may be an unexplained exception with greater blood prolactin in winter ^54^, but results from different genders and populations seem inconsistent. Wehr found that long scotophases were associated with longer nocturnal elevations of prolactin among both men and women, suggesting that human prolactin may be higher when melatonin is higher ^55, 56^. One study of afternoon prolactin found slightly higher prolactin during winter in premenopausal females, but patients with winter depression (either unipolar or bipolar) had much lower prolactin than controls in both summer and winter ^57^. Any causal role for prolactin in mood swings seems uncertain.

## More studies relating thyroid homeostasis to mood

During the middle of the 20 ^th^ century, Richter demonstrated that lesions of the rat pituitary-thyroid axis produced periodic cycles of activity resembling rapid mood cycles. Richter pointed out the relationship of thyroid impairments to the manic-depressive mood cycles that had been described in early clinical studies ^58, 59^. Despite this hint, generations of psychiatrists studying thyroid effects on mood may have been frustrated or misled by the poor correlations between peripheral blood indices of thyroid function and mood, which may result from poor correlations between the T3 concentrations in the blood versus the T3 concentrations in the basal hypothalamus that might be the crucial determinant of mood symptoms. There is a variety of evidence for subclinical hypothyroidism in unipolar and bipolar depression ^60^, and the antidepressant response to sleep deprivation is related to the TSH response and to variations in the activity of circulating TSH that are attributable to the degree of sialylation ^61^.

Nevertheless, much evidence has accumulated that functional brain hypothyroidism is associated with depression and with bipolar and rapid-cycling manic-depressive symptoms ^46, 59, 62– 66^. Because functional brain hypothyroidism may not be indicated by standard blood thyroid indices reflecting thyroid regulation outside the brain, seemingly supraphysiologic oral doses of thyroxine may be required to benefit mood ^67^. It may be necessary to add T3 to T4 supplementation ^67^. Besides mood affects, elevated hypothalamic T3 increases appetite, which might help counter the loss of appetite associated with depression ^68^. Moreover, there are now several genetic polymorphisms in thyroid-regulation genes reported to influence depression and mania, perhaps through influences on hypothalamic T3 regulation ^69^. A *DIO2* polymorphism is associated with the lifetime incidence of major depression ^70^. Two other *DIO2* polymorphisms have been related to poor mental health ^71^. There are several other polymorphisms known to influence thyroid metabolism, though their possible role in hypothalamic T3 regulation seems inadequately explored ^72, 73^. A *TEF* promoter SNP has been reported to be associated with depression ^74^. Also, humans have a very common single nucleotide polymorphism labeled rs1321108 in the So1 binding site of the *TSHB* promoter, altering it and possibly influencing the promoter functions of EYA3 and SIX1. Contemporary genome-wide association studies (GWAS) have not confirmed that these polymorphisms (SNPs) are associated with major depression or bipolar disorder. However, in bipolar disorder and separately in major depressions, whole genome expression studies have observed reduced DIO2 RNA expression in a frontal basal brain area (P=0.008), not confirmed by overall meta-analysis; DIO3 was increased in the same study and in the same area (P=0.005) in bipolars but not in meta-analysis whereas DIO3 was decreased in major depression (P<0.02); the major T3 receptor in the hypothalamus, THRA, was reduced in that area (P=2.32E-06) and in an adjacent site in major depression and increased among bipolars in two assays but not in meta-analysis; TEF is increased in frontal cortex of bipolars by meta-analysis (P<0.05), especially in fronto-basal cortex, but not in MDD ^75, 76^. In summary, there are now scattered clinical and genetic findings indicating that photoperiodic PT control of hypothalamic T3 levels might interact with other aspects of thyroid regulation to contribute to causal pathways both for bipolar mania and for development of depression.

Bright light is known at times to trigger mania ^77^, whereas darkness therapy is an effective acute anti-manic treatment ^78, 79^. Because morning bright light immediately suppresses melatonin, early morning light should lead to increased EYA3 production, with consequent elevations of hypothalamic T3, whereas darkness would lower hypothalamic T3. Peripheral hyperthyroidism may produce mental disturbances that sometimes resemble mania, so perhaps hypothalamic excess T3 is largely responsible for the manic phenotype ^62^. If a bipolar person had a genetic tendency towards circadian phase delay, in the Spring as the days grow longer and dusk occurs later in the evening, the EYA3 peak might rise later in the day, while an early dawn might mask (suppress) melatonin and thus disinhibit the EYA3 peak. Hypothalamic TSH and T3 might then become excessively elevated, leading to an April-June peak in mania. Another factor is short sleep or a night without sleep, that tends to predict mania ^80, 81^, and may often involve increased light exposure at night. A consistent finding is that airplane passengers travelling from west to east (so that they would be exposed to daylight before their normal melatonin offset) tend to become manic, whereas travelers from east to west (so that darkness might retard melatonin offset) are more likely to become depressed ^82, 83^. The east-going air travel effect is consistent with the antidepressant effect of advancing sleep ^84^. Nevertheless, since bright light or air travel do not make most people manic at any time of year, a more complicated interaction of factors is likely involved.

## Sleep and photoperiodic mechanisms

Sleep restriction (wake therapy) may have dramatic antidepressant effects and may sometimes trigger mania ^85^. Indeed, a single night of sleep loss often seems to trigger the onset of mania ^81^. The antidepressant effect of sleep restriction seems partly (but not entirely) mediated by light at night ^86– 88^. Whether sleep loss influences PT production of TSH apart from light effects on melatonin appears to be unknown. We have located no data regarding effects of sleep deprivation upon TSH and T3 in the 3 ^rd^ ventricle CSF. Remaining awake past a normal nocturnal bedtime produces a sudden increase in blood TSH ^89^. With normal human sleep, blood TSH falls abruptly at sleep onset, though the extent to which this is due to darkness or to some aspect of sleep itself is uncertain.

A combination of partial sleep restriction (“wake therapy”), phase-advancing the timing of sleep (and awakening), and morning bright light have an enhanced and almost immediate antidepressant action, but there are insufficient comparative controlled trials to prove that this innovative triple combination is more antidepressant than bright light treatment alone ^84, 87^. One may speculate that the triple combination treatment could further limit melatonin inhibition of *EYA3* transcription and therefore lead to more enhanced hypothalamic TSH and T3 synthesis. In certain models, morning light exposure by itself produces circadian rhythm phase advances accompanied by temporary abbreviation of the duration of melatonin secretion ^20, 90, 91^, though this abbreviation was not documented in our own studies demonstrating light-induced advances of melatonin ^92^.

## Winter depression, other depression, and photoperiodic control of mood

The mechanism of winter depression (Seasonal Affective Disorder or SAD) can be understood theoretically from the photoperiodic mechanisms that we have reviewed. The long nights of winter prolong nocturnal melatonin secretion and delay the morning melatonin secretion offset, particularly among SAD patients (with possible gender inconsistencies) ^93, 94^. Winter depressives tend to have circadian-phase-delayed melatonin as well as perhaps an expanded duration of secretion ^94, 95^, either of which can cause delayed melatonin offset. A delayed offset of melatonin would inhibit pars tuberalis EYA3 and TAC1 production among winter depressives just as in laboratory rodents, thus inhibiting hypothalamic T3 production. This theory is supported by the distinct antidepressant effectiveness of early morning bright light which suppresses late night melatonin ^91, 96^ as well as by the antidepressant effectiveness of morning propranolol and atenolol, beta blockers that can also suppress melatonin ^96– 98^. This theory is likewise supported by the high prevalence of depression among people with delayed sleep phase disorder, as explained in Figure 1 ^99^.

It has been shown that depressed people tend to display circadian rhythm phase delays at all times of year, most notably in the melatonin offset ^100– 104^. A tendency towards eveningness (mild symptoms of circadian delay) is associated with lack of remission of depression ^105^. For bipolar manic-depressives, eveningness (e.g., sleep phase delay) is a characteristic trait partly independent of mood state ^106^. Bipolar patients in remission display an actigraphic sleep interval that is longer (though with more midsleep awaking and poorer sleep efficiency ^107^, perhaps suggesting longer time-in-bed rather than increased actual total sleep time), and this predicts depression relapse ^108^. Further, when depressed, bipolars are more likely to experience long sleep than unipolar depressives ^109^, which might indicate a particular tendency of the morning oscillator and melatonin offset to delay among bipolars. In one study, bipolars had later peaks of nocturnal melatonin than controls, but melatonin offsets were not recorded ^104^. Further, there is evidence from small samples of patients that bipolars display a long cellular free-running circadian cycle in their fibroblasts in tissue culture ^110, 111^, presumably of genetic origin. If a similar trend towards a longer cellular circadian period were found in SCN cells, it would contribute to a delayed melatonin circadian rhythm and delayed offset. Depression is most often associated with insomnia, but bipolar depression is also commonly associated with long or excessive sleep. Long sleep and delayed melatonin offset are associated ^112^. It is possible that simply because they spend a longer time in bed, both people with insomnia and long sleepers may delay their first substantial morning light exposures ^113^, thus allowing a delayed melatonin offset to mask their EYA3 peak transcription.

A specific genetic contribution to phase delay may arise from polymorphisms in *CACNA1C*, a calcium channel component which mediates light-induced shifts of circadian phase ^114^, perhaps through effects on both GSK3B and also on CREB (CREB mediates light stimulation of the SCN) ^115^. *CACNA1C* is one of the loci most strongly associated with bipolar disorder in GWAS studies (as well as less strongly associated with schizophrenia and major depressive disorder) ^116^. A polymorphism in *ASMT*, the last gene in the melatonin synthesis pathway, is associated with circadian phase delay and perhaps with inadequate melatonin synthesis (factors that combined might augment or inhibit EYA3), and with depression and bipolar disorder ^117, 118^. In addition, there have been quite a few reports of genetic variants associated with affective disorders in genes participating in circadian oscillator regulation, but we feel there has as not been adequate replication of these findings, including our own ^99, 119– 124^.

There is evidence that depressed people experience below-average daytime illumination overall compared to the population as a whole ^125, 126^. Depression may result at least in part from light deficiency, especially morning light deficiency, whether from the winter season, circadian phase delays, long sleep, or various social, behavioral, or occupational factors. Moreover, depressive symptoms are treated successfully by morning bright light treatment as well as by manipulations of sleep and circadian phase at any time of year ^84, 127^. Data from a population survey suggested that adults who were more depressed spent longer times in bed, possibly because they experienced more light at night ^113^. Even though light at night when found in ordinary households is associated with depression, the reported light intensities do not seem bright enough to substantially reduce total nocturnal melatonin production ^113, 128, 129^.

Some theoretical difficulties should be acknowledged. There is evidence that a minority of patients with winter depression may have advanced melatonin in reference to their sleep times ^130^, at least as measured by the dim-light melatonin onset. There might be phase-advance as well as phase-delay variants of nonseasonal depression and bipolar illness as well ^131^ that possibly might result if the EYA3 peak becomes more phase-advanced than does melatonin offset. Seasonal summer depression is more difficult to explain, but might arise from people staying indoors in hot weather, thus prolonging nocturnal melatonin secretion, although direct hypothalamic suppression of thyroid function by summer heat might also be involved.

## Complexities in control of mood

Given our proposed theory of winter depression, it is difficult to explain Spring seasonal peaks in hospitalizations for depression, suicide, and mania e.g., April and May in the northern hemisphere. Some authorities have hypothesized that these Spring peaks are due to prolongation or exacerbation of depressions that begin as winter depression, or rebounds therefrom, but there are few specific data to support this view. A genetic trend towards phase delay in melatonin offset may explain how depressions due to inadequate pars tuberalis EYA3 and low hypothalamic T3 might occur at any time of year, but a genetic predisposition to delay does not explain why symptoms should peak near or just after the Spring equinox. We may speculate that a genetic tendency to delay could exacerbate effects of the spring transition to longer days. Spring lengthening of days results in a delay shift of the evening oscillator caused by later sunsets, which we combine with the “Daylight Savings” advance in the time standard that influences time in bed. The combined effect makes sunset suddenly much later by our adopted time standard. As days grow longer in Spring, a balancing advance shift of the morning oscillator might be anticipated due to earlier dawns, but melatonin offset was not found earlier in summer among normal urban subjects, perhaps partly because of the shift in time standard ^132^. In contrast, melatonin offset was found to be earlier in summer than winter among winter depressives, with some differences between men and women ^132^. An endogenous tendency to delay among depressives could make advance of the morning oscillator in Spring especially indolent, especially if combined with a relative unresponsiveness of the morning oscillator to phase-advancing light exposures. Numerous studies indicate some asymmetry of responses to light stimuli causing swifter light-stimulated delay phase shifts versus advances as photoperiods vary ^90, 114, 133^. We may speculate that perhaps the spring transition to a shorter scotophase combined with the Daylight-Savings time reference could produce a delay in melatonin offset despite an earlier EYA3 peak, accentuating the morning melatonin masking of EYA3 with consequent depression. On the other hand, in a rat model, light-induced phase advance may temporarily suppress pineal n-acetyltransferase (NAT), thus suppressing melatonin production ^90^. These competing processes promoting possible increased or decreased melatonin masking of EYA3 in the Spring might produce the paradoxical peaks of both depression and mania at about the same season, depending on various factors influencing susceptibility in a diverse population. Whether Spring tends to promote depression or mania may depend on the extent to which the later sunset delays melatonin offset more than the EYA3 peak, despite an earlier dawn. An unexplained mystery is how the peak of EYA3—seemingly about 12 hours after dark--is controlled and synchronized.

## Rapid cycling and circadian oscillator desynchronization

A feature of some bipolar syndromes that is particularly hard to understand is the appearance of rapid cycling, that is, episodes of depression and/or mania which come and go at least four times a year. In extreme cases, mania and depression may alternate every few days or even every other day ^81, 134^. Halberg hypothesized that such mood swings could be caused by a free-running desynchronized circadian rhythm with a cycle longer than 24 hours, so that its peak drifted later each day relative to the 24-hour light-dark cycle ^135^. One might consider this an “external desynchronization” model, that is, where all of the body’s internal rhythms might remain synchronized to each other, but they might free-run progressively later and later, beating in and out of phase with the external environment, particularly, its light-dark cycle. Both Kripke, Wehr and their colleagues tried to document such non-24-hour rhythms among rapid-cycling patients, but apart from a very few intriguing examples that did not seem fully persuasive, they had little success ^78, 131, 136, 137^.

On the other hand, Wehr reported clear demonstrations that repeated 48-hour sleep wake cycles are at times observed among bipolar patients, in association with 48-hour cyclic manic-depressive symptoms ^81^. Wehr attributed these 48-hour cycles to an “internal desynchronization” model wherein the temperature rhythm and many other circadian rhythms remained synchronized to the 24-hr environment, but the period of sleep-wake (and some associated rhythms) decelerated so much as to double cycle length and produce 48-hour rhythms. Overt symptoms depended on when critical intervals of these two sets of rhythms were in or out of phase. In temporal isolation and cave experiments, circadian rhythms of core temperature and related functions have at times been observed to free-run with cycle periods of about 25.0 hr., while the sleep-wake rhythm might internally desynchronize to cycles as long as 36 hr. or even 48–50 hr ^138, 139^. Because in these internal desynchronization models, sleep-wake cycles tend to be much more unstable and generally slower, debate has emerged about whether sleep-wake should be considered a “weak” non-linear circadian oscillator or alternatively a homeostatic relaxation oscillator that should not be classified as circadian. In any case, the internal desynchronization observed in isolation studies has not generally been recognized to cause mood disturbances in cave and isolation experiments, despite a few severe psychoses reported in such experiments. The absence of daylight and the dark surrounding of cave environments might have been a protective antimanic factor. The isolation and cave studies did prove that cycles of alternating long and short sleep or even 48-hr sleep-wake cycles could arise as a consequence of circadian internal desynchronization ^140^.

In rats, overall SCN firing is higher in the day (when the animals mainly sleep) than at night, and the duration of multiunit neuronal firing is longer in long photoperiods. However, within either the photophase or scotophase, firing is somewhat higher in wake and in REM than in SWS ^141^. SCN metabolic activity is likewise higher in the day and further increased by light exposure ^142^. In rats, when core and shell were internally desynchronized by 22-hour light-dark cycles, both the SCN core and shell remained associated with slow-wave sleep, but REM sleep and body temperature were more exclusively associated with activity of the shell neurons ^143^. It would appear from the responses to phase-shifting light-dark cycles, considering the two-process model of sleep-wake control, that the more-directly-light-responsive core is associated better with the homeostatic aspect of sleep-wake regulation, whereas the shell is better associated with the circadian modulation of sleep wake. In diurnal mammals, also, SCN firing tends to be higher during the day times when these animals tend to be awake, but whether firing of SCN core or shell augments or suppresses sleep among diurnal animals is unknown to us, and therefore, it would be uncertain how to relate the SCN core and shell division to internal desynchronization in humans.

## Circadian oscillator bifurcation

**Figure 2.**
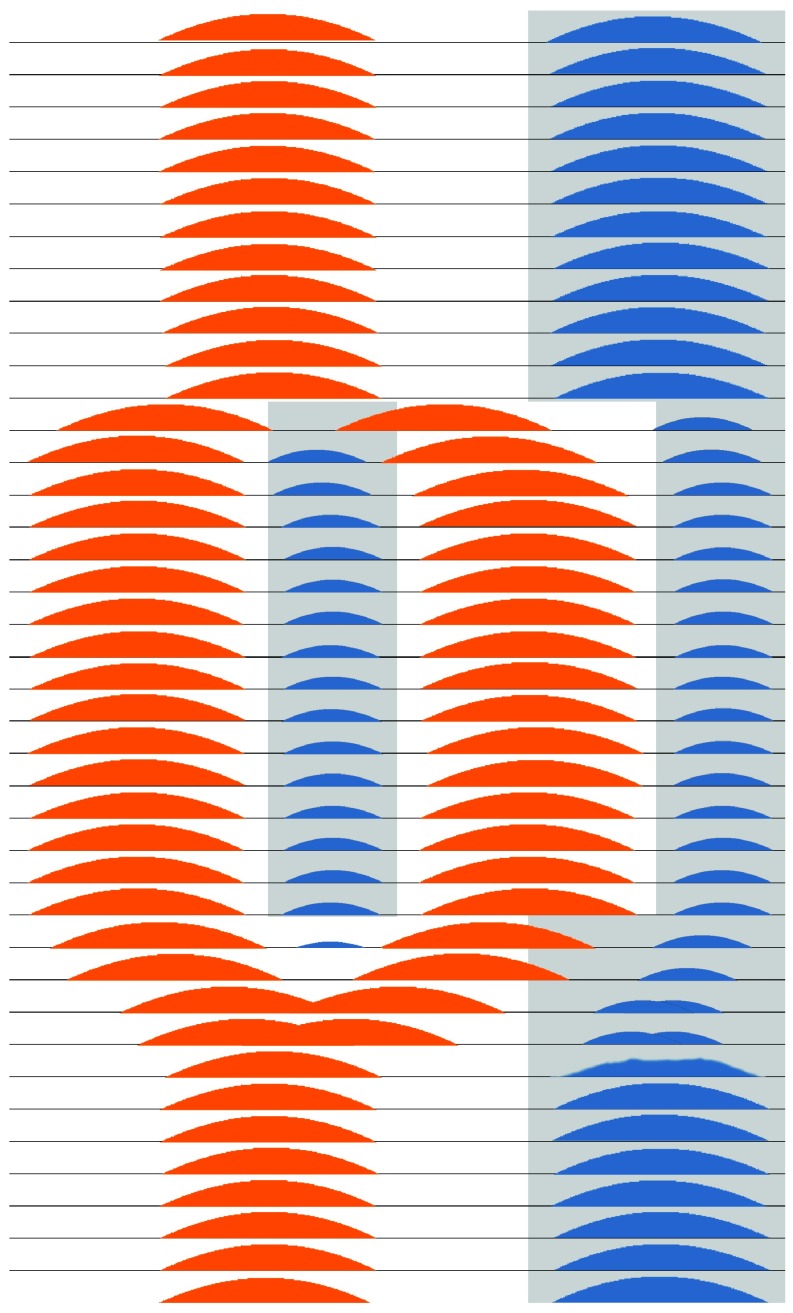
Theoretical schematic of circadian bifurcation in humans. In this diagram, each line of the ordinate represents a 24-hour day and the abscissa represents the 24 hours within that day. The grey shading depicts very dim light or darkness, whereas the white background represents daylight and artificial light. The light-dark cycle is modelled as commencing with LD16:8 and transitioning in the middle days to LDLD8:4:8:4, with return to LD16:8 in the final days. The orange shading represents SCN multiunit neuronal firing that gradually splits apart and bifurcates into two antiphase patterns of firing during LDLD8:4:8:4, representing two distinct populations of coupled SCN neurons. During LD16:8, firing might be spread out over a longer interval in the light than is shown, but there may be insufficient data to model the pattern of neuronal timing more exactly. After return to LD16:8 or to continuous darkness (DD), the two components of neuronal firing gradually fuse together again. The blue regions represent melatonin secretion during the dark intervals. Suppressed by neuronal firing and light suppression, it is plausible that melatonin secretion would be partly or completely inhibited during the transitions from LD16:8 to LDLD8:4:8:4 and back again, during which melatonin secretion would bifurcate and then fuse again. These patterns are theoretical, because the transitions of neuronal firing and melatonin secretion from an LD pattern to a bifurcating LDLD pattern and back again have never been observed simultaneously in detail, certainly not in a diurnal mammal.

Perhaps we may gain further insight into mechanisms that could trigger mania by considering circadian rhythm bifurcation, which is the division of the circadian rhythm into two components, with the two peaks being separately entrainable. Circadian research has developed certain laboratory models that “bifurcate” nocturnal rodent activity into two circadian components (bouts of activity) about 12 hours apart from each other. These two activity bouts can be entrained in a stable manner by a special light-dark cycle consisting of two photophases (light intervals) and two scotophases (dark phases) within each 24 hours (for example, light-dark-light-dark hours abbreviated as LDLD7:5:7:5) ^144^. A large set of studies utilizing Syrian hamsters, Siberian hamsters, and mice have demonstrated stable entrainment of bifurcated scotophase activity bouts, body temperature peaks, and melatonin peaks ^145^. Taken together, these studies lead to the hypothesis that LDLD entrainment bifurcates the neural oscillators in the SCN into two or more components, each driving activity, body temperature, and melatonin secretion. Bifurcated-rhythm hamsters develop and maintain summer gonadal size and presumed reproductive fertility ^146^, perhaps because the duration of each bout of melatonin secretion is brief (as in the short nights of summer). The bifurcated activity components and bifurcated melatonin secretion in the scotophases represent the control of two independently-entrainable circadian pacemakers which are yet mutually coupled, and which will fuse into a single component if the bifurcated photophase is withdrawn ^71, 147^. In this model, the circadian bifurcation seems to result from two different populations of neurons in the SCN that assume almost opposite phases, though the two bifurcated SCN populations appear to be bilaterally symmetrical ^148, 149^. It should be emphasized that the two scotophases and two photophases per 24 hr day do not need to be absolutely symmetrical, especially once the bifurcation has occurred. When the bifurcated photoperiod is replaced by constant dark and the bifurcated activity bouts rejoin each other, the melatonin secretion components presumably also fuse. The two activity bouts can be recoupled either by the day-scotophase activity component delaying or by the day component advancing in reference to the night-scotophase activity component ( Figure 2) ^71^.

Conceivably, LDLD bifurcation into two circadian oscillator components produces two peaks in EYA3 transcription, neither of which is well-suppressed by melatonin, thus promoting increased PT TSH production and increased tanycyte production of T3.

The attainment of bifurcated circadian activity cycles by LDLD lighting cycles and the phase-shifting effects of light in general can be enhanced in nocturnal rodents by very dim illumination during the dark scotophases, for example, 0.005 lux ^147^. This would be less than 1% of bright moonlight and too dim to suppress melatonin. Since a dim-light scotophase causes the duration of the rodent nocturnal activity phase (alpha) to expand, it has been inferred that dim light weakens the coupling between separate circadian neuronal populations ^71, 147, 150, 151^. Coupling refers to the mutual influence of one oscillator on another. Conceivably, even dim light might suppress LHX1, a transcription factor (known to be suppressed by bright light) that mediates expression of VIP and the AVP receptor AVPR1A, thus impairing coupling of SCN neurons ^152^.

In humans, Worthman and Melby have found that in the tropical and subtropical environments in which, for the most part, our species developed, daytime napping is quite pervasive, especially near the middle of the day ^153^. One wonders if the bifurcated activity patterns which are observed in hot climates in equatorial regions, where the heat of the day leads to mid-day sun avoidance, might also mimic a bifurcated LDLD laboratory photophase and the resultant bifurcated circadian organization. However, the common human daytime sleep episodes usually described do not constitute half of 24-hour sleep. In primitive surroundings, illumination intensities are substantial during daytime sleep, unlike the dim scotophase in the rodent circadian bifurcation model. When people in equatorial climates or in summer at high latitudes remain awake in the cooler night, often using artificial light, or among night shift workers, a somewhat-bifurcated bright-light photophase might be combined with dim light exposure during the scotophases.

We do not know much about human circadian responses to bifurcated sleep conditions such as those suggested by napping. We are unfamiliar with any evidence that bifurcated melatonin secretion may be produced or that the two sleep episodes come to represent two independently-entrainable circadian oscillators. Unfortunately, we have virtually no information concerning what levels of light at night might produce phenomena in humans similar to the dim night light effects promoting circadian bifurcation in hamsters and mice, if indeed this scotophase effect occurs in humans at all, and we have no definite data concerning what conditions might produce true bifurcated circadian oscillators among humans. Our own pilot studies attempting to induce bifurcated melatonin rhythms with LDLD cycles produced only a few bimodal melatonin peaks of unequal amplitude, and we are unsure if longer exposure to LDLD or addition of dim light to the dark scotophases might have led to more convincing bifurcation. Although it has been asserted that irregular sleep cycles may induce bipolar relapses and mania ^154^, there is no evidence that specific conditions producing bifurcated sleep patterns in humans predispose to depression or mania. Moreover, it is likely that interactions of genetic variations with environmental factors are required to trigger major mood disorders.

## Phase jumping caused by light and singularity

Related to circadian rhythm bifurcation, another peculiar phenomenon called “phase jumping” should be considered. When bright light compresses the effective primary scotophase excessively (e.g., to around 4 hours in some nocturnal rodents, LD20:4), and an alternative secondary scotophase is made available, the originally nocturnal activity bout may “jump” to a relative antiphase orientation in the newly opened scotophase ^147^. This phase jumping is likewise augmented by dim light during the scotophase, and might result partly from light-suppression of LHX1, VIP, and AVPR1A. Possibly during the very long summer days that occur at higher latitudes and with certain patterns of artificial light, phase jumping might be triggered. Similarly, it is conceivable that severe sleep restriction (e.g., no more than 4 or 5 hours in bed) would trigger human phase jumping if coupled with other permissive conditions (e.g., a daytime retreat with darkness or very dim lighting).

Using mice bred with a PER1 or PER2-bound luciferase, SCN slices can be monitored *in vitro* over time. Luciferase luminescence is then a marker of the molecular circadian clock phase of individual SCN neurons. In slices from mice housed in the dark or in LD12:12 (that is a photoperiod of 12 hours light and 12 hours dark), the peak times of PER2-luciferase activity do not differ more than a few hours in various SCN regions, nor is the neuronal timing determined simply by core-shell VIP-AVP or rostral-caudal parameters ^21, 27^. As the duration of the photophase is incrementally increased, the phase distribution of SCN neurons broadens substantially, until in LD20:4, a population of mostly-core neurons and a population of mostly-shell neurons are 6–12 hr out-of-phase with each other ^21, 155^, somewhat resembling rodent LDLD bifurcation experiments. Released into DD (continuous darkness), the mutual coupling of these two neuronal pacemaker populations pulls them back into alignment, either through relative advances or delays of the core-like population in reference to the shell-like population. After exposure to such atypical photoperiods, the coupling of the two groups of pacemaker neurons might undergo a full 360° circadian phase rotation in reference to each other ^155^. Thus, over many days, one pacemaker component might steadily delay (or advance) relative to another, reminiscent of the non-24-hour components and internal desynchronization previously hypothesized to trigger rapid mood cycling in humans.

Another phenomenon which might possibly be involved in mania is a complex of light pulses that may drive a circadian system to its singularity point, apparently stopping the clock ^156^. It is possible that an anti-phase orientation of the core and shell could at times produce an appearance of SCN singularity while both core and shell remain inversely oscillatory ^157^. Stopping or severely attenuating SCN rhythmicity could have profound consequences for brain function, e.g., memory ^158^. Bright constant light may also suppress circadian activity rhythms in rodents ^159^. Human circadian rhythms are occasionally driven through an apparent singularity by phase-shifting stimuli ^160^, but in the presence of a synchronizing environment, the circadian rhythms appear to recover after a few days. It is conceivable that an interval of singularity in at least one portion of the SCN, e.g., the core, is an element in sudden switches into mania.

## Core and shell circadian bifurcation, melatonin bifurcation, and mania

To recapitulate, both a bifurcated photophase, e.g., LD7:5:7:5 or a very long photophase, e.g., LD20:4 can evidently phase shift two SCN neuronal populations towards a near-antiphase SCN pacemaker organization from which a 360° phase rotation between the two pacemakers might evolve. In rodents, dim light during the scotophases enhances this bifurcation. The anti-phase orientation of two SCN neuronal populations could result in internal circadian desynchronization somewhat resembling the observations in temporal isolation, cave experiments and LD phase shifts, but appearing much less overt than the full external circadian desynchronization hypothesized by Halberg for rapid mood cycles of several days ^135^. Indeed, we do not know exactly how internal circadian desynchronization or resynchronization (reorganized phase relationships among SCN components) might best be documented among humans. The best clue comes from experiments in which a bifurcated LDLD cycle produced bifurcated locomotor activity in Siberian hamsters that was associated with two temporally dissociated episodes per day of melatonin production, one melatonin secretion interval seemingly coupled to the SCN core oscillator and the other to the shell ^28, 145^. Note, behavioral and reproductive data from related studies indicated that these short intervals of melatonin production would be associated with gonadal fertility ^146^, permitting an inference that hypothalamic TSH and T3 were produced at long-day concentrations or greater.

Collecting blood, saliva, or urine samples for melatonin every few hours from severe manics has been so challenging that until recently we could locate no substantial body of round-the-clock observations of melatonin from manics which might reveal if a bifurcated rhythm or a shifted phase relationship among distinct SCN oscillator components are likely to be associated with mania. Remarkably, an outstanding group of investigators has now overcome the challenges of collecting 24-hour saliva samples from manics for assaying melatonin. Their exciting new evidence shows that during acute mania, bipolars indeed produce two antiphase separated peaks of melatonin secretion, one at night and one in the day, much like the melatonin secretion of bifurcated Siberian hamsters ^145, 161^. The investigators suggested that the bifurcated peaks in melatonin secretion might be due to disruption of coupling between SCN oscillators. There had been previous observations of two largely-merged peaks of melatonin as well as possibly some rare unmerged double-peaks even among normal subjects ^162^, or a possible small daytime peak among occasional winter depression patients ^96^, but we do not know of situations apart from mania in which fully-separated and relatively equal and symmetrical antiphase melatonin peaks have been observed in humans. We can speculate that bifurcated melatonin excretion could prove a valuable marker of a bifurcated antiphase orientation of human SCN neuronal populations, and perhaps this antiphase SCN organization is the specific circadian disorder of mania.

Since in normal humans, waking activity is highest in the day, but melatonin is highest at night encompassing the hours of sleep, we might expect that among manics with bifurcated melatonin secretion, bifurcated locomotor activity or bifurcated sleep-wake patterns might also be observed. The trail-blazing study of human melatonin in manics displaying two peaks as described above did not record sleep-wake or activity ^161^, but some informative wrist activity plots in mania were published by Wehr’s group ^81, 163^. In the plots from Wehr’s observations, we could not discern any persuasively bifurcated activity rhythms—on the other hand, brief daytime cessations of activity in these plots prevent us from being certain that a bifurcated activity rhythm did not occur among the manics recorded.

Another finding related to this theory of bifurcated SCN pacemaker components in mania comes from the following clinical observations. Rapid-cycling bipolars seem less likely to suffer relapse if bright light treatment is given near midday ^164^, a time when bright light might tend to reverse a bifurcation between SCN pacemaker components.

We have an interesting model of potential circadian sleep bifurcation among human shift workers. On the one hand, it has been asserted that circadian behavioral irregularities such as those produced by shift work schedules promote mania ^154^. On the other hand, mania is not generally noted among shift workers, although it is often convenient for night shift workers to divide their sleep between several hours in the morning just after the night shift and an additional 1–3 hours in late afternoon or evening before going to work. So far as we know, bifurcated melatonin rhythms have not been described among such night shift workers, perhaps because many shift their melatonin rhythms little from their day-work pattern. Also, the dim lighting during most night shifts might be protective. To the extent that attempts to use bright light to promote alertness during night shift work are effective in shifting melatonin secretion rhythms, such lighting might increase the risks of triggering mania or depression. Evidence for possible bifurcation in sleep-wake has been reported from certain circadian isolation studies, but there was no evidence for mania in these studies, in which melatonin was not assessed ^165^.

## Complex light stimuli: possibilities triggering circadian bifurcation and mania

Now we may synthesize hypotheses of how mania could result from a disorder of photoperiodic regulation. From the poor sleep and often early awakening of manics, it appears that some aspect of circadian regulation becomes disordered during mania. The triggering of mania by a single night’s sleep loss or midsleep awakening or perhaps more chronic sleep compression or phase shifts ^81, 83, 166^ raises the question of whether a sudden internal phase shift between two SCN component oscillators may produce a switch into mania. If a melatonin-related SCN oscillator component became delayed well past dawn, perhaps because of sleep loss and use of artificial lighting late at night, then morning light might further delay that component past noon, triggering internal circadian desynchronization or bifurcation. Dim light at night might facilitate the sudden phase shift, since a person suffering se **v**ere sleep disturbance for any reason is likely to turn on artificial lighting irregularly at night, and this may weaken coupling of SCN component circadian oscillators. We currently have no evidence base from which to judge what intensities and timings of light might be most likely to produce internal desynchronization or altered phase of circadian oscillator components in humans, but it does appear that bright morning light perceived at or before the usual time of awakening might contribute ^77, 91^. Likewise, since sleep restriction in the second half of the night seems almost as effective as whole-night sleep deprivation in its antidepressant effects ^167^, it seems likely that sleep in this second half of the night is most critical to preventing mania. The often-discussed shortened sleep and elevated mood experienced by Scandinavians near the summer solstice might be a modest human replica of the LD20:4 response, or the impressive peak in violent suicides in Greenland at about the same season might be an even more dramatic model ^10, 168^. At present, we have only one strong study showing that the circadian system is actually bifurcated during mania (as indicated by melatonin). The difficulties of collecting such data must be acknowledged, but perhaps we know better now what measurements are needed.

A seeming paradox arises from our hypothesis that mania may arise from excessive phase delays of an oscillator component within the SCN, in part due to genetic tendencies to delay, since mania is more often described as a condition of early awakening and phase advance ^161^. Perhaps the explanation is that should bright daytime light cause an SCN component to delay more than 180°, it becomes advanced from the perspective of the other component. The mutual coupling of SCN oscillator components might be expected to resolve any transient internal desynchronization within a few days, but perhaps bright light exposures both soon after awakening and again past mid-wake would stabilize persistent mania, just as LDLD lighting can prevent bifurcated circadian oscillations from resolving among hamsters. Likewise, it becomes logical that either bright light at some critical time of day or round-the-clock darkness would tend to resolve antiphase malsynchronization of the two SCN oscillator components. The empirical observations that mania may resolve when a patient is treated with the delaying drug lithium or with a dark environment ^79^ may be consistent with these speculations. Perhaps the process of resolution of mania can be monitored by studying the evolution of the two peaks of melatonin in mania.

## Conclusion and needs for future research

To conclude, we have proposed that photoperiodic mechanisms, interacting with inadequate or untimely illumination and a genetic tendency for phase delay, produce depression. Among bipolars, we propose that combined with genetic susceptibilities, abnormal bright light illumination patterns trigger mania by producing internal desynchronization and perhaps bifurcation of SCN circadian oscillator components, thus leading to photoperiodic malregulation and excess hypothalamic T3 production. Data supporting a photoperiodic mechanism triggering depression are already quite strong: both evidence for delayed melatonin offsets and delayed awakenings among depressed patients and evidence that forcing the melatonin offset earlier (with bright light treatment or propranolol) is antidepressant. Data supporting circadian bifurcation as the cause of mania do not extend beyond the seminal observation of a bifurcated melatonin excretion pattern in one study of manics ^161^ and some support among other scattered and uncertain clinical observations. The hypotheses presented have many limitations including missing elements of the proposed neurobiologic mechanisms, an insufficient evidence base, some apparent inconsistencies with available data, and insufficient testing of predictions. These hypotheses are presented as a call for much further study and testing of predicted effects, both among laboratory animals and among consenting human volunteers.

Here are some key areas for future research:

- Further observations of bifurcated 24-hour melatonin secretion among manics are needed, extended by descriptive longitudinal data during the development and remission of mania. Likewise, data on sleep-wake, activity, and core temperature are needed to correlate with melatonin changes during the evolution of mania.
- Blood measurement of PT TSH ^47^ in humans could confirm impaired secretion of PT TSH in depression, a positive response to light, and excessive PT TSH in mania.
- Long-term longitudinal descriptive monitoring of bipolar patients (becoming increasingly practical with the growing mass-market for health-monitoring actigraphic wrist bands) should be initiated to try to identify what lighting patterns trigger depression and mania, and how the consequent activity patterns evolve. Considering the ethical obligation to “do no harm,” we do not recommend attempts to trigger depression or mania experimentally, but in the long run, observational research may lead to testable preventive interventions.
- More clinical trials are needed to optimize bright light treatment timing, sleep-wake phase-advance, and sleep restriction combinations in relieving depression. Likewise, more clinical trials are needed to clarify what manipulations of light or darkness or melatonin agonists might cause mania to remit.
- Systematic dose-response studies should define what levels (and color-spectrum) of dim light might facilitate loosening of SCN component oscillator coupling in humans, with possible resultant increased melatonin secretion durations, facilitated phase-shifting, and perhaps facilitated circadian bifurcation of sleep and locomotor activity behaviors in humans.
- Systematic experiments should search for photoperiod manipulations which can produce circadian bifurcation in humans, and measure the related endocrine and mood responses.
- Shift workers with bifurcated sleep patterns should be re-examined to see if bifurcated melatonin secretion results, and if this correlates with mood disorders. Studies of various transmeridional air travel effects may also clarify the roles of varying light exposure patterns.
- Systematic experiments should examine if very long photoperiods, e.g., 20:4, when coupled with an inserted phase of darkness or dim illumination, can produce phase-jumping in humans, with consequences in activity, sleep wake, melatonin secretion, and mood. Both among humans and laboratory animals, data are needed as to whether phase jumping produces bifurcated melatonin secretion patterns.
- We would like to see testing of the hypothesis that the circadian bifurcation produced by LDLD skeleton photoperiods produces bifurcated large EYA3 peaks in PT and consequent increased third ventricle TSH and T3. Perhaps this could be tested in a diurnal mammal, possibly using microdialysis of TSH or T3 in the third ventricle CSF near PT. Possibly *in-vivo* MR spectral imaging could be an alternative to microdialysis.
- Blood measurement of PT TSH ^47^ could be useful to assess effects of circadian bifurcation in rodents.
- We would like to understand the molecular mechanism controlling the timing of the morning peak in PT EYA3, absent melatonin inhibition.
- We would like clarification of how PT regulation of hypothalamic T3, which does impact TRH secretion, interacts with the peripheral homeostatic regulation of thyroid metabolism.
- We would like to see further study of the SCN neurophysiology related to LDLD-induced circadian bifurcation, including clarification of core-shell and anterior-posterior SCN functional differentiation, clarification of the SCN neuroanatomical structures, and exploration of which SCN-efferent neurotransmitters such as AVP and VIP mediate bifurcated secretion of melatonin when it occurs. Likewise, clarification of the contrasts and overlap between the evening-morning oscillator models and core-shell oscillator models is needed.
- Similarly, we would like to see clarification of the neuronal firing patterns in the core and shell regions of the SCN after LDLD bifurcation as they relate to the component circadian oscillators in both nocturnal and diurnal rodents.
- We would like more data on the effects of LDLD circadian bifurcation in rodents on reproductive endocrine functions.
- We would like to see how LDLD-induced circadian bifurcation influences rodent behavioral models of depression and mania.
