# Supplementary material for: Photoperiodic and circadian bifurcation theories of depression and mania
Source: F1000Res. 2015 May 6;4:107. [Version 1] doi: 10.12688/f1000research.6444.1 (PMC4490783; doi:10.12688/f1000research.6444.1)
Supplement: Peer review history

# Peer review: Referee response for version 1

**DOI:** 10.5256/f1000research.6915.r9288

**Published:** 2015-07-02

**Authors:** Robert D. Levitan

**Affiliations:** 1 Centre for Addiction and Mental Health, University of Toronto, Toronto, ON, Canada

This is a thoughtful and timely review of an important topic in mood disorders i.e. the circadian regulation of mood and its relevance to the understanding and treatment of major mood disorders. Despite significant efforts over many decades, and incremental improvements in the treatment of depression and bipolar disorder, many patients do not respond to standard treatments. At a clinical level it is apparent that the vast majority of patients with major mood disorders have a significant disruption in one or more aspects of circadian regulation whether it be sleep, activity rhythms, social behavior, energy regulation, hormonal function etc. The current paper adds significantly to the literature in both proposing new ways of thinking about circadian dysregulation and mood and proposing a series of studies that follow from the models proposed. The proposed studies also exemplify well the new Research Domain Criterion strategy to understanding complex pathology.

Overall, the paper has a strong emphasis on basic mechanisms, often at a molecular level, which can be difficult to follow at times. It might be of interest for the authors to balance this approach with a consideration of the possible evolutionary/adaptive significance of the mechanisms under consideration as this might provide further context for their arguments. For example, what might be the advantage to a particular species of having the ability to bifurcate circadian rhythms? Or do the authors propose that this bifurcation only occurs in pathological situations or when the system is stressed beyond its normal physiological control?

In terms of specific mechanisms, while the current paper emphasizes melatonin studies at many points, it can be argued that the neurotransmitter dopamine is as important to daytime activity rhythms and arousal as melatonin is to sleep rhythms and physiology at night. Accumulating evidence suggests that there are several ways by which melatonin and dopamine interact and modulate one another, including basic circadian rhythmicity (reviewed by Zisapel, 2001). Furthermore, there is a large body of work demonstrating dopaminergic dysfunction in mood disorders and bipolar disorder in particular. It may be that the best way forward for future work on circadian physiology and mood is to consider these two systems as one integrated system and to understand their interplay in the regulation of mood. How this might relate to the proposed hypotheses and research program might be of great interest going forward.

I have read this submission. I believe that I have an appropriate level of expertise to confirm that it is of an acceptable scientific standard.

# Peer review: Referee response for version 1

**DOI:** 10.5256/f1000research.6915.r8564

**Published:** 2015-06-01

**Authors:** Klaus Martiny

**Affiliations:** 1 Psychiatric Centre Copenhagen, Copenhagen University Hospital, Copenhagen, Denmark

This comprehensive review of seasonality, mood, circadian regulation, and their inter-relatedness is of great value for the creation of new hypotheses in the field and gives impetus for a number of new studies. The title is appropriate even though the paper touches on a number of additional subjects such as separate morningness and eveningness oscillators and the regulation of thyroid function in relation to mood. The abstract covers the content of the paper. Many theories are presented and perhaps the red thread is sometimes a little difficult to follow in the narrative. Of the many ideas presented the hypothesis regarding separate oscillators for morningness and eveningness is of immediate interest. It should be investigated how this phenomenon is reflected in the rating scales used to assess chronotype and time-dependent preferences. Do we need separate scales for morningness and eveningness? In the quest for understanding the higher prevalence of suicide in springtime authors do not include the widely accepted psychological concept that for a person with persistent depression, the contrast of seeing other persons elation and increased energy in springtime, could contribute to the phenomenon. This raises a more general point: why is the interaction or cascading effect between cognition (thought and emotion) and circadian physiology not included in our understanding of the regulation of mood? A few critical points: Authors uses the word sleep restriction for sleep deprivation (wake therapy). Sleep restriction in the sense of a continuous reduction in sleep time does not induce an antidepressant effect. The effect of sleep deprivation depends on sleep abstinence for a substantial part or the whole of a single night AND the timing of recovery sleep days. Also the effect of sleep deprivation probably relates to the relation between sleep and the activity of the raphe nuclei on the brain-stem and this should be mentioned. Figure 1 is rather busy and could be simplified to enhance readability. On page 5 the effect of darkness as an antimanic treatment is defined as “effective”. At this point we should probably regard the evidence of dark therapy, however interesting, as preliminary (and difficult to carry out). The statement, also on page 5, that the effect of sleep deprivation is partly caused by light, is not quite substantiated by the one reference dealing with this issue. Finally, references should be checked for content in relation to their relation to issues discussed in the paper with a more critical attention to the quality of the studies (case reports and pilot studies).

I have read this submission. I believe that I have an appropriate level of expertise to confirm that it is of an acceptable scientific standard.

# Response

**Published:** 2015-06-04

**Authors:** Daniel F. Kripke

**Affiliations:** University of California, San Diego, USA

We appreciate Dr. Martiny's thoughtful review as well as his important contributions to light treatment of depression. Some of the interesting issues he raises deserve further discussion.

As Dr. Martiny points out, the concept of chronotype and the usual methods of measuring chronotype do not recognize the partially-independent phases of morning-oscillator and evening-oscillator components. However, the relative timing of evening and morning components seems crucial to the photoperiodic responses in animals and apparently in humans. Although our presentation emphasizes the melatonin offset, the dim light melatonin onset or DLMO has become popular as a more-easily-measured phase marker. It is likely that the DLMO is correlated with the timing of the EYA3 peak in pars tuberalis. As demonstrated in much animal work and to some extent at http://www.ncbi.nlm.nih.gov/pmc/articles/PMC1988787/  Figure 6, melatonin onsets and offsets may have slightly different phase-response curves. Factor analysis of morningness-eveningness scales has likewise suggested the presence of partly independent morning-related and evening-related factors, so we agree that there could indeed be value in generating separate morning and evening subscales from existing chronotype questionnaires. 

As Dr. Martiny pointed out, the dramatic antidepressant response that he has successfully employed clinically depends on sleep abstinence for a substantial part of the night and also upon the timing of recovery sleep. The term sleep restriction is commonly used for milder reductions of sleep duration continuing over weeks or longer. Cognitive-behavioral treatments of insomnia that include an element of sleep restriction seem to be antidepressant, e.g., see http://www.ncbi.nlm.nih.gov/pmc/articles/PMC3353033/ , though it is unclear whether time-in-bed or EEG sleep curtailment or earlier morning light exposure is most determinative.  The mood effects of long-term mild sleep restriction need more clarification, especially for those with long sleep. Although there is a large literature decrying the effects of sleeping too little, more mortality and as much morbidity (including depression) is associated with unusually long reported sleep durations. 

As Dr. Martiny mentioned, many of the ideas assembled in our review are based on meager experimental evidence, so more study is clearly needed.
